# Malan syndrome in a patient with 19p13.2p13.12 deletion encompassing *NFIX* and *CACNA1A* genes: Case report and review of the literature

**DOI:** 10.1002/mgg3.997

**Published:** 2019-10-01

**Authors:** Fernanda T. Bellucco, Claudia B. de Mello, Vera A. Meloni, Maria Isabel Melaragno

**Affiliations:** ^1^ Department of Morphology and Genetics Universidade Federal de São Paulo São Paulo Brazil; ^2^ Department of Psychobiology Universidade Federal de São Paulo São Paulo Brazil

**Keywords:** 19p13.2 microdeletion, *CACNA1A*, Malan syndrome, *NFIX*, overgrowth disorder

## Abstract

**Background:**

Malan syndrome is a recently introduced overgrowth disorder described in a limited number of individuals. Haploinsufficiency and also point mutations of *NFIX* gene have been proposed as its leading causative mechanism, however, due to the limited number of cases and different deletion sizes, genotype/phenotype correlations are still limited.

**Methods:**

Here, we report the first Brazilian case of Malan syndrome caused by a 990 kb deletion in 19p13.2p13.12, focusing on clinical and behavioral aspects of the syndrome.

**Results:**

The patient presented with macrocephaly, facial dysmorphisms, hypotonia, developmental delay, moderate thoracolumbar scoliosis, and seizures. The intellectual and behavioral assessments showed severe cognitive, language, and adaptive functions impairments. The 19p deleted region of our patient encompasses *NFIX*, *CACNA1A*, which seems to be related to a higher frequency of seizures among individuals with microdeletions in 19p13.2, and 15 other coding genes, including *CC2D1A* and *NACC1,* both known to be involved in neurobiological process and pathways.

**Conclusion:**

Deletions involving *NFIX* gene should be considered in patients with overgrowth during childhood, macrocephaly, developmental delay, and seizures, as well as severe intellectual disability.

## Graphical abstract

Malan syndrome is an overgrowth disorder described in a limited number of individuals and, due to the limited number of cases and different deletion sizes, genotype/phenotype correlations are still limited. We report a new case of Malan syndrome caused by a 990 kb deletion in 19p13.2p13.12, and revise the previous reports discussing possible genotype–phenotype correlations within the deleted region.

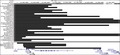

## 1 INTRODUCTION

Malan syndrome (OMIM #614753), also named Sotos‐like syndrome or Sotos syndrome 2, characterized by overgrowth, facial dysmorphism, intellectual disability and behavior problems, is a recently introduced clinical condition described in a limited number of individuals. So far, descriptions of patients in the literature comprise cases of haploinsufficiency of *NFIX* gene, due to chromosomal microdeletions in 19p13.2 region (Auvin, Holder‐Espinasse, Lamblin, & Andrieux, 2009; Bonaglia et al., 2010; Dolan et al., 2010; Dong et al., 2016; Hino‐Fukuyo et al., 2015; Jezela‐Stanek et al., 2016; Jorge, Silva, Águeda, Dória, & Leão, 2015; Karmarkar, Amarillo, & Larsen, 2014; Klaassens et al., 2015; Kuroda et al., 2017; Lyon et al., 2015; Lysy et al., 2009; Malan et al., 2010; Natiq et al., 2014; Nimmakayalu et al., 2013; Priolo et al., 2018; Shimojima et al., 2015; Welham et al., 2015), and also point mutations in *NFIX* (Gurrieri et al., 2015; Jezela‐Stanek et al., 2016; Klaassens et al., 2015; Lu et al., 2017; Malan et al., 2010; Martinez et al., 2015; Oshima et al., 2017; Priolo et al., 2012, 2018; Rai, Narayanan, & Phadke, 2018; Yoneda et al., 2012).

Here we report a new case of Malan Syndrome caused by a 990 kb deletion in 19p13.2p13.12. This is the first Brazilian case and it highlights clinical and behavioral aspects of the syndrome.

## 2 CLINICAL REPORT

The patient was the first child of nonconsanguineous and healthy parents after a preterm and uneventful pregnancy. His measurements, corrected for birth at 34 1/7 weeks, were as follows: birth weight 2.645 g (0.37 *SD*), birth length 47 cm (1.9 *SD*), and head circumference 35 cm (3.9 *SD*). At 20 months of age, his height was 87 cm (1 *SD*), weight was 12 kg (0.50 *SD*), and head circumference was 50 cm (1.72 *SD*). He showed dysmorphic features including relative macrocephaly, long face, frontal bossing, frontal flat hemangioma, down‐slanting palpebral fissures, deep‐set eyes, long eyelashes, open mouth appearance, pointed chin, long hands, slender habitus, and severe hypotonia.

**Figure 1:**
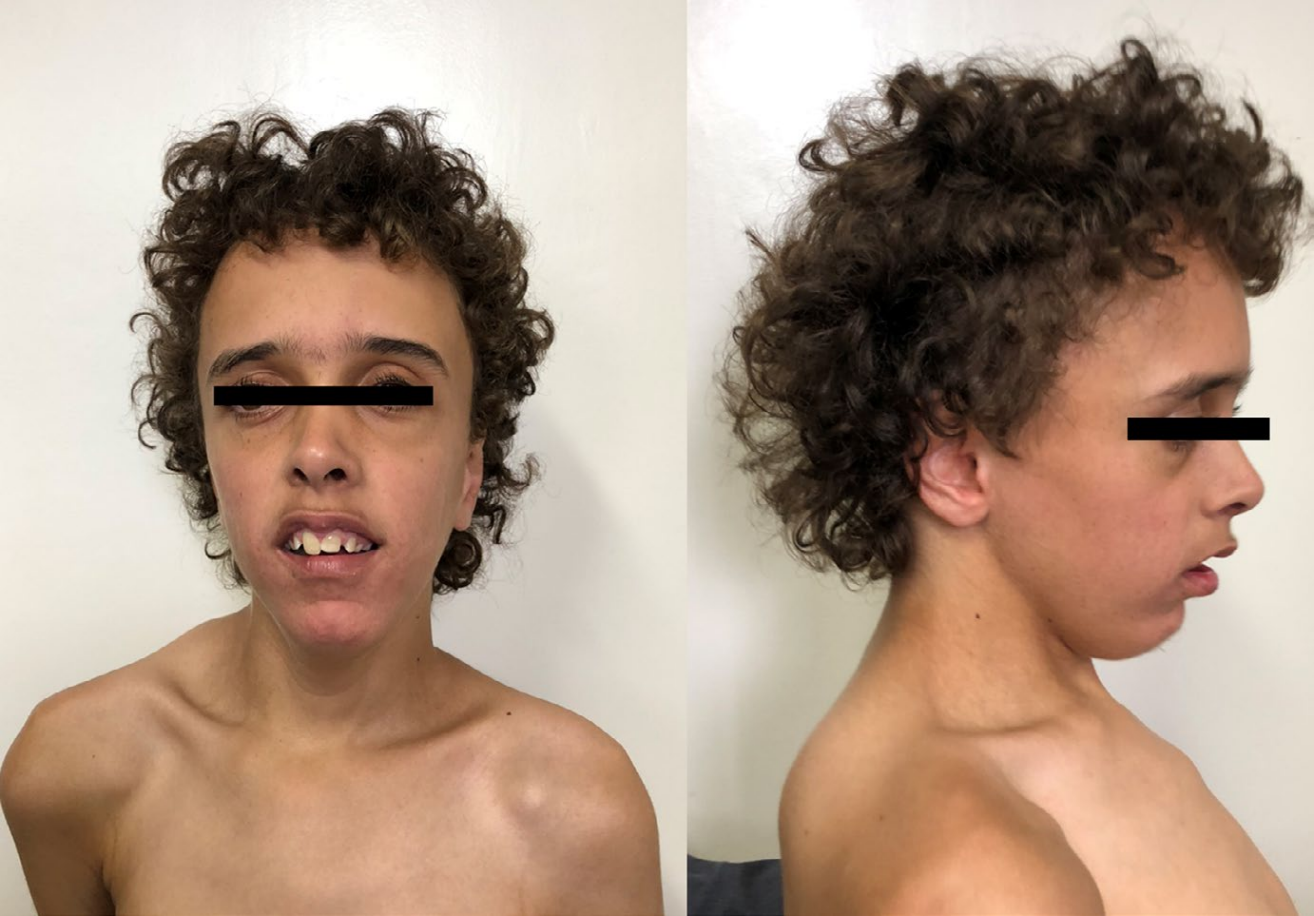
Patient at 19 years and 7 months of age showing macrocephaly, long face, frontal bossing, down‐slanting palpebral fissures, deep‐set eyes, long eyelashes, anteverted nares, open mouth appearance, and pointed chin

The first seizure episode was at 10 months of age and it developed into tonic seizures that were controlled initially by phenobarbital, and then by clonazepam plus valproate. Follow‐up EEG showed bilateral frontotemporal sharp waves, and a cranial MRI‐scan showed a reduction of the periventricular white matter with a compensatory dilation of the lateral ventricles and the third ventricle. At 9 years and 8 months of age, his measurements were: height 135 cm (−0.24 *SD*), weight 35 kg (0.73 *SD*) with a BMI‐for‐age 19.2 (1.08 *SD*) and head circumference 56.5 cm (>3 *SD*). In addition, he presented moderate thoracolumbar scoliosis, pectus carinatum, exotropia, and pale retina. At 19 years and 7 months of age (Figure 1), his measurements were: height 155 cm (−3.01 *SD*), weight 45 kg (−3.45 *SD*) with a BMI‐for‐age 18.7 (1.79 *SD*) and head circumference 57.5 cm (2 *SD*).

## 3 INTELLECTUAL AND BEHAVIORAL ASSESSMENTS

The patient was submitted to an intellectual performance assessment, which included the Brazilian version of the Wechsler Abbreviated Intelligence Scale—WASI (Trentini, Yates, & Heck, 2014) and The Vineland Adaptive Behavioral Scale—VABS‐II (Sparrow, Cicchetti, & Balla, 2005). Additionally, parents answered the Child Behavior Checklist—CBCL (Achenbach & Resorta, 2001), by which presence of behavioral problems was investigated. A neuropsychologist conducted the assessment in individual sessions and appropriate rooms of a neurodevelopmental outpatient clinic from the Universidade Federal de São Paulo, Brazil.

At the time of assessment, the patient was 19 years old. Analysis of parents’ answers to the VABS‐II indicated low performance (standard‐score < 40) in all adaptive domains: Communication (receptive, expressive, and written), Daily Life Skills (personal, domestic, and community), Socialization (interpersonal relationships, play and leisure time, and coping skills) and Motor Skills (Table S1). He presented a limited understanding of verbal instructions and communicated only by vocalizations and a few meaningful gestures. Therefore, an accurate assessment of intellectual quotient (IQ) was not possible. Quantitative analysis of parents’ answers to the CBCL did not indicate the presence of any internalizing (e.g., anxiety or depression) or externalizing (e.g., aggression) behavioral problems at clinical levels (t‐score < 65).

## 4 CYTOGENETIC AND MOLECULAR STUDIES

This research was approved by the Ethics Committee of UNIFESP. Informed consent for clinical and genetic analyses was obtained from the patient's parents.

G‐banding karyotype was performed on peripheral blood lymphocyte cultures, according to standard procedures and revealed normal results. Array assay using the CytoScan 750K Array (Affymetrix, Santa Clara, CA, USA) showed a 990 kb 19p13.2p13.12 deletion. The final result was given as 46,XY. arr[GRCh37/hg19] 19p13.2p13.12(13068720_14053305)×1.

## 5 DISCUSSION

**Figure 2:**
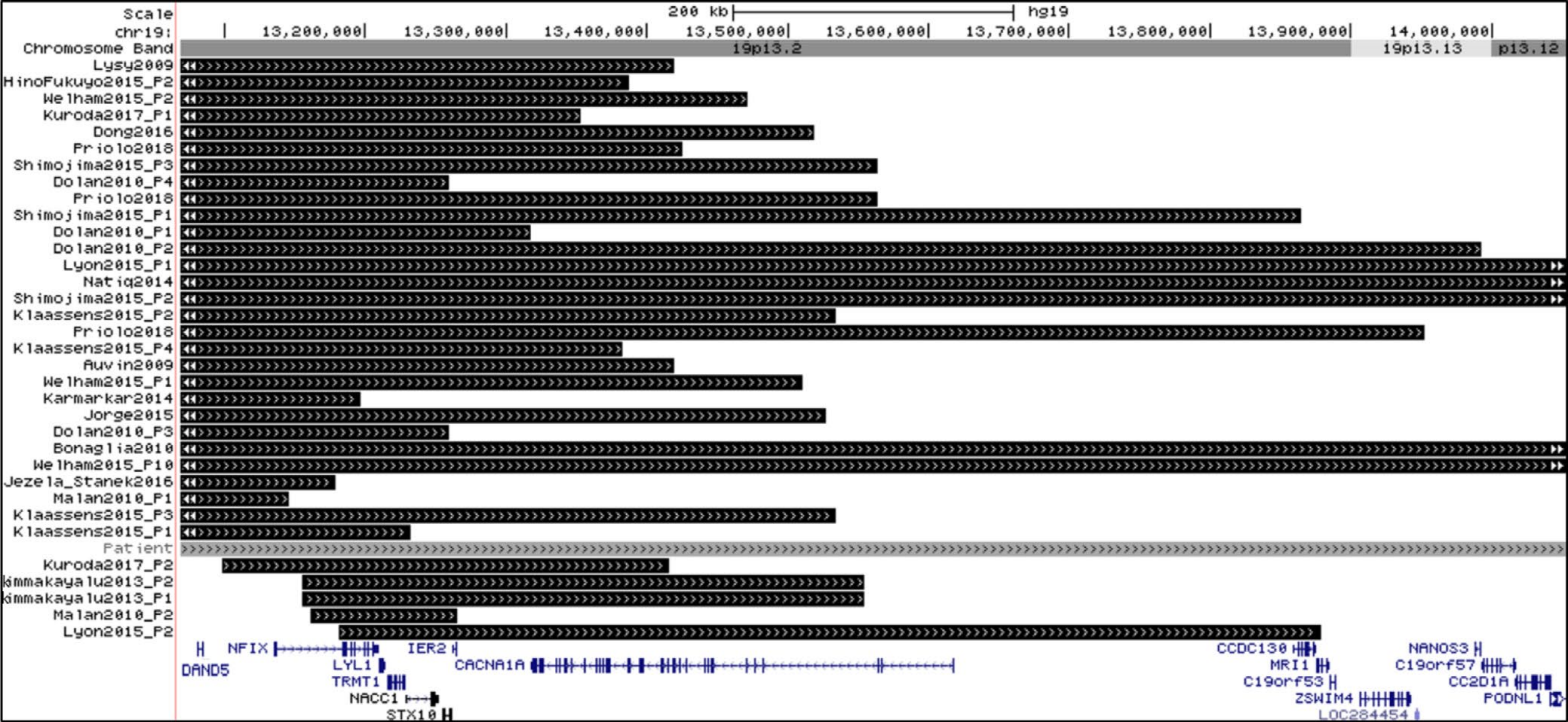
Genome map of the overlapping deletions of our patient (gray bar) and previously reported cases (black bars), and known genes in the chromosome 19p13 region. The patients` rearrangements were organized based on their initial deletion coordinates. Data were uploaded to UCSC genome browser, NCBI Build 37, February 2009, hg19 Assembly (http://www.genome.ucsc.edu)

We identified a 990 kb deletion including the *NFIX* gene in a patient with Malan syndrome. *NFIX* haploinsufficiency or point mutations, clustered mostly in exon 2 (Priolo et al., 2018), are the leading causative mechanism in Malan syndrome (Gurrieri et al., 2015; Klaassens et al., 2015; Malan et al., 2010). *NFIX* (OMIM * 164005), a member of the nuclear factor I family of transcription factors, is essential for normal brain and skeletal development and *Nfix* deficiency in mice leads to brain malformations including ventriculomegaly and partial agenesis of the corpus callosum (Driller et al., 2007; Malan et al., 2010). To date, together with the present study, 35 patients were described with Malan syndrome phenotype, all of them presenting deletions involving *NFIX* gene, with variable breakpoints (Auvin et al., 2009; Bonaglia et al., 2010; Dolan et al., 2010; Dong et al., 2016; Hino‐Fukuyo et al., 2015; Jezela‐Stanek et al., 2016; Jorge et al., 2015; Karmarkar et al., 2014; Klaassens et al., 2015; Kuroda et al., 2017; Lyon et al., 2015; Lysy et al., 2009; Malan et al., 2010; Natiq et al., 2014; Nimmakayalu et al., 2013; Priolo et al., 2018; Shimojima et al., 2015; Welham et al., 2015) (Figure 2).

Apart from overgrowth and intellectual disability/global developmental delay, the most consistent clinical feature of patients with Malan syndrome is facial dysmorphism. Priolo et al. (2018) refined genotype–phenotype correlations in individuals with Malan syndrome and found that all patients presented with typical facial features that include a long or triangular face (which become more elongated with age), prominent forehead, depressed nasal bridge, deep‐set eyes, down‐slanting palpebral fissures, short nose with anteverted nares and upturned tip, long philtrum, small mouth that is often held open, with a thin upper vermillion in a cupid bow shape, an everted lower lip, and a prominent chin. Our patient presented with the main facial dysmorphic features of patients with Malan syndrome and also, moderate thoracolumbar scoliosis, pectus carinatum, exotropia, and pale retina. According to Priolo et al. (2018), visual impairments are common and skeletal anomalies are frequent in patients with Malan syndrome, especially a slender habitus, scoliosis, pectus carinatum, or excavatum, which occur at varying frequencies.

Despite being considered an overgrowth syndrome, neither weight nor length at birth are above the 2 *SD* in the majority of patients (Priolo et al., 2018). Comparing all the patients described in the literature who present 19p13 microdeletion encompassing the *NFIX* gene, 48.5% (15/31) presented with postnatal overgrowth in height, and in only 9% (2/22) the birth length is above 2SD (Table S2). Macrocephaly, on the other hand, seems to be an important and more frequent clinical feature in patients with 19p13 microdeletion. Among patients with *NFIX* haploinsufficiency, we found that 36% (9/25) present head circumference above 2 *SD* at birth and 58% (18/31) in the postnatal period (Table S2). Our patient presented macrocephaly at birth and during infancy, but at 19 years and 7 months of age, his head circumference was 57.5 cm (2 *SD*), with a height of 155 cm (−3.01 *SD*), suggesting a relative macrocephaly. It is important to observe that moderate scoliosis also compromises his height.

The clinical features observed in individuals with deletions of *NFIX* and a variable number of other genes do not show significant differences in comparison with individuals who present *NFIX* point mutations. The main difference seems to be related to the frequency of seizures/epilepsy and EEG abnormalities among individuals with microdeletions (Kuroda et al., 2017; Priolo et al., 2018), which may be explained by the presence of a contiguous gene disorder. Located 109 kb from *NFIX*, *CACNA1A* has been associated with such increased prevalence of seizures (Auvin et al., 2009; Marangi et al., 2012; Natiq et al., 2014; Priolo et al., 2018). *CACNA1A* gene (OMIM *601011) encodes a voltage‐dependent calcium channel subunit expressed in neuronal tissue and mutations in this gene have been associated with epilepsy (Kors et al., 2004; Zamponi, Lory, & Perez‐Reyes, 2010) and chronic neurological disorders (Ducros et al., 2001; Ophoff et al., 1996; Wan et al., 2011). Of 27 individuals reported with deletion encompassing *NFIX* and *CACNA1A* genes, including our patient, 12 presented with seizures (Auvin et al., 2009; Bonaglia et al., 2010; Hino‐Fukuyo et al., 2015; Kuroda et al., 2017; Lyon et al., 2015; Natiq et al., 2014; Nimmakayalu et al., 2013; Priolo et al., 2018; Shimojima et al., 2015). On the other hand, four of eight patients have been reported with seizures and 19p13.2 deletions not involving *CACNA1A* (Dolan et al., 2010; Jezela‐Stanek et al., 2016; Klaassens et al., 2015; Table S2). Since 12 of 27 (44,5%) cases presenting seizures and *CACNA1A* deletions is not significantly different from 4 of 8 (50%) cases presenting seizures and non‐*CACNA1A*‐containing deletions, we could not confirm, based on the cases described so far, that *CACNA1A* haploinsufficiency is the only factor responsible for the increased prevalence of seizures among patients with 19p deletion.

Previous studies describing patients with Malan syndrome reported intellectual disability varying from moderate to severe levels, as well as behavioral profiles marked by anxiety symptoms (Priolo et al., 2018). Welham et al. (2015) studied behavioral characteristics of a group of 10 patients with 19p13.2 microdeletions. The authors found a high frequency of autism spectrum disorders characteristics, repetitive, and challenging behaviors (such as aggression and self‐injury) and they concluded that the characteristics might be associated with 19p13.2 microdeletions. However, in only three cases, the deleted region included *NFIX*.

Our patient showed severe overall cognitive and adaptive deficits, with an expressive language repertoire restricted to few words and meaningful gestures. During the clinical interview, the mother reported signs of anxiety, but only in new situations or unfamiliar contexts. The clinical geneticist also identified signs of anxiety in routine medical consultations. On the other hand, different from Welham et al. (2015) findings, the parents’ answers to the CBCL did not indicate the presence of internalization or externalization, or even autistic‐like, behavioral problems.

Besides *NFIX* and *CACNA1A* genes, the 19p deleted region of our patient encompasses 15 other coding genes (Figure 2). Among them, deletions of *CC2D1A* gene (OMIM *610055), which regulates the expression of serotonin receptors in neuronal tissue and have been associated with non‐syndromic intellectual disability (Basel‐Vanagaite et al., 2006), and *NACC1* gene (OMIM *610672), which encodes a protein that works as a transcriptional regulator and is associated with neurodevelopmental disorder characterized by epilepsy, cataracts, feeding difficulties, and delayed brain myelination (Schoch et al., 2017), might be able to contribute to the severe cognitive and adaptive functions impairments found in our patient, since they are involved in neurobiological pathways and processes. However, it should be noted that *CC2D1A* is a recessive intellectual disability gene, while *NACC1* is associated with a dominant disease caused by a recurrent missense variant, so detailed studies about the involvement of this genes, as well as the other genes located in the deleted 19p region of our patient, besides *NFIX*, could accurately define the genotype–phenotype correlations linked to 19p deletions.

In conclusion, we suggest that deletions involving *NFIX* gene should be considered in patients with overgrowth during childhood, macrocephaly, developmental delay, and seizures, as well as severe intellectual disability. Our cytogenomic, clinical, and behavioral assessment data contribute to a better understanding of the syndrome for an accurate diagnosis, prognosis, and genetic counseling of Malan syndrome patients.

## DATA AVAILABILITY STATEMENT

The data that support the findings of this study are available from the corresponding author upon request.
