# Supplementary material for: Malan syndrome in a patient with 19p13.2p13.12 deletion encompassing NFIX and CACNA1A genes: Case report and review of the literature
Source: Mol Genet Genomic Med. 2019 Oct 1;7(12):e997. doi: 10.1002/mgg3.997 (PMC6900369; doi:10.1002/mgg3.997)
Supplement: Supplementary file 1 [file MGG3-7-e997-s001.docx]

**Table S1:** Characteristics of adaptive behavior

| **Subdomain/Domain** | *Raw Score* | *v-Scale Score* | *Standard Score* | *Percentile* |
| --- | --- | --- | --- | --- |
| **Communication** | Sum=5* | | 26 | <1 |
| Receptive | 12 | 1 |  |  |
| Expressive | 12 | 1 |  |  |
| Written | 0 | 3 |  |  |
| **Daily Live Skills** | Sum=4* | | 25 | <1 |
| Personal | 10 | 1 |  |  |
| Domestic | 0 | 2 |  |  |
| Community | 0 | 1 |  |  |
| **Socialization** | Sum=7* | | 34 | <1 |
| Interpersonal Relations | 21 | 2 |  |  |
| Play and Leisure Time | 4 | 1 |  |  |
| Coping Skills | 0 | 4 |  |  |
| **Motor Skills** | Sum=12* | | 49 | <1 |
| Gross Motor Skills | 50 | 8 |  |  |
| Fine Motor Skills | 12 | 4 |  |  |

*Sum of subdomain v-scale scores (Mean=15; Standard Deviation=3).
