# Supplementary material for: Malan syndrome in a patient with 19p13.2p13.12 deletion encompassing NFIX and CACNA1A genes: Case report and review of the literature
Source: Mol Genet Genomic Med. 2019 Oct 1;7(12):e997. doi: 10.1002/mgg3.997 (PMC6900369; doi:10.1002/mgg3.997)
Supplement: Supplementary file 2 [file MGG3-7-e997-s002.docx]

**Table S2:** Summary of clinical findings in patients with deletions including *NFIX* gene.

|  | | | **Present study** | **Lysy**  **2009** | **Auvin 2009** | **Bonaglia 2010** | **Malan**  **2010** | **Dolan 2010** | **Nimma-kayalu**  **2013** | **Karmarkar**  **2014** | **Natiq 2014** | **Shimojima 2015** | **Klaassens 2015** | **Jorge**  **2015** | **Hino-Fukuyo**  **2015** | **Lyon**  **2015** | **Welham**  **2015** | **Jezela-Stanek 2016** | **Dong 2016** | **Kuroda 2017** | **Priolo 2018** | **Total** |
| --- | --- | --- | --- | --- | --- | --- | --- | --- | --- | --- | --- | --- | --- | --- | --- | --- | --- | --- | --- | --- | --- | --- |
| Gender | |  | M | F | M | M | 1M/1F | 1M/3F | 2F | F | F | 3F | 2M/2F | F | M | 2F | 1M/2F | F | M | M/F | 2M/1F | 35 |
| Prenatal | Birth weight >2SD | | - | - | - | - | 1/2 | 3/4 | NA | NA | - | 0/3 | 1/4 | - | NA | 0/2 | NA | - | + | 0/2 | 0/2 | 6/27 |
|  | Birth length >2SD | | - | - | - | - | 1/2 | NA | NA | NA | - | 0/3 | 0/4 | - | NA | 0/1 | NA | + | - | 0/2 | 0/2 | 2/22 |
|  | OFC >2SD | | + | - | - | - | 1/2 | 3/3 | NA | NA | - | 1/3 | 0/4 | - | NA | 1/1 | NA | - | - | 0/2 | 2/2 | 9/25 |
| Postnatal | Height >2SD | | - | - | + | - | 2/2 | 3/4 | 1/2 | + | + | 1/3 | 2/4 | + | NA | 0/2 | NA | - | - | 1/2 | 1/3 | 15/31 |
|  | OFC >2SD | | - | - | + | - | 2/2 | 4/4 | 2/2 | + | + | 1/3 | 1/4 | + | NA | 0/2 | NA | + | - | 1/2 | 2/3 | 18/31 |
| Development | Intellectual disability/GDD | | + | + | + | + | 2/2 | 4/4 | 2/2 | + | + | 3/3 | 4/4 | + | + | 2/2 | 3/3 | + | + | 2/2 | 3/3 | 35/35 |
|  | Hypotonia | | + | + | + | + | 2/2 | 3/4 | 2/2 | + | + | 3/3 | 2/4 | + | NA | NA | NA | + | + | 2/2 | 3/3 | 26/29 |
| Craniofacial | Long or triangular face | | + | - | + | + | 2/2 | 4/4 | 2/2 | NA | + | 3/3 | 2/4 | + | NA | 1/1 | NA | - | + | 0/2 | 3/3 | 23/29 |
|  | Prominent forehead | | + | + | + | + | 2/2 | 4/4 | 2/2 | NA | + | 3/3 | 4/4 | + | NA | 1/1 | NA | + | + | 2/2 | 3/3 | 29/29 |
|  | Depressed nasal bridge | | - | + | - | - | 1/2 | 1/4 | 0/2 | NA | - | 1/3 | 1/2 | NA | NA | 0/1 | NA | - | + | 2/2 | 1/3 | 9/26 |
|  | Deep set eyes | | + | - | + | - | 1/2 | 2/4 | 0/2 | NA | + | 2/3 | 1/2 | NA | NA | 0/1 | NA | - | + | 2/2 | 3/3 | 15/26 |
|  | Down-slanting palpebral fissures | | + | - | NA | + | 1/2 | 1/4 | NA | NA | + | 2/3 | 3/4 | NA | NA | 0/1 | NA | - | NA | 0/2 | 2/3 | 12/24 |
|  | Short nose | | + | + | + | - | 2/2 | 3/4 | 0/2 | NA | + | 2/3 | 2/2 | NA | NA | 0/1 | NA | - | + | 1/2 | 1/3 | 16/26 |
|  | Upturned nasal tip/anteverted nares | | + | - | + | + | 2/2 | 1/4 | 0/2 | NA | + | 3/3 | 2/2 | + | NA | 0/1 | NA | + | + | 1/2 | 1/3 | 17/27 |
|  | Small mouth | | - | - | + | + | 1/2 | 1/4 | 1/2 | NA | - | 2/3 | 2/2 | NA | NA | 0/1 | NA | + | + | 1/2 | 0/3 | 12/26 |
|  | Thin upper vermillion/cupid bow | | + | - | + | + | 1/2 | 2/4 | 1/2 | NA | + | 3/3 | 2/2 | NA | NA | 0/1 | NA | + | + | 2/2 | 2/3 | 19/26 |
|  | Everted lower lip/open mouth appearance | | + | - | + | - | 2/2 | 4/4 | 0/2 | NA | + | 1/3 | 2/2 | NA | NA | 1/1 | NA | + | + | 1/2 | 3/3 | 19/26 |
|  | Pointed/prominent chin | | + | - | + | + | 2/2 | 3/4 | 2/2 | NA | + | 1/3 | 2/2 | NA | NA | 0/1 | NA | - | + | 2/2 | 2/3 | 19/26 |
| Eyes | Vision impaired | | + | + | - | NA | 2/2 | 4/4 | 2/2 | NA | + | 0/3 | 3/4 | + | NA | 1/1 | 1/3 | + | + | 2/2 | 2/3 | 23/31 |
| Skeletal | Slender habitus | | + | NA | - | NA | 2/2 | 1/4 | 1/2 | NA | - | 2/3 | 0/4 | + | NA | NA | NA | + | - | NA | 3/3 | 12/25 |
|  | Kyphoscoliosis | | + | NA | - | + | 1/2 | NA | 0/2 | NA | - | NA | 1/4 | NA | NA | NA | NA | - | - | 1/2 | 3/3 | 8/19 |
|  | Craniosynostosis | | - | + | - | - | NA | NA | - | NA | - | - | NA | - | NA | 2/2 | NA | - | NA | NA | NA | 3/11 |
|  | Pectus excavatum/carinatum | | + | NA | - | + | 2/2 | NA | 1/2 | NA | - | NA | 3/4 | + | NA | NA | 1/3 | - | + | 1/2 | 3/3 | 15/23 |
|  | Large long hands | | + | NA | - | NA | 2/2 | 2/2 | 1/2 | NA | - | NA | 1/1 | NA | NA | NA | NA | + | - | 1/2 | 3/3 | 12/17 |
|  | Advanced bone age | | NA | NA | + | NA | 2/2 | 1/1 | NA | NA | + | NA | 1/1 | + | NA | NA | NA | + | NA | 1/1 | 2/3 | 11/12 |
|  | Joint laxity | | + | NA | - | NA | 2/2 | NA | 1/2 | NA | - | NA | NA | NA | NA | NA | NA | + | - | 0/2 | 2/3 | 7/14 |
| Brain | MRI abnormality | | + | + | - | - | 1/2 | 3/4 | 1/2 | + | + | 2/3 | NA | **-** | **-** | 1/2 | NA | - | + | 2/2 | 1/2 | 16/27 |
| Seizures | Deletion involving *CACNA1A* | | + | - | + | + |  | 0/1 | 1/2 |  | + | 2/3 | 0/3 | - | **+** | 1/2 | 0/3 |  | - | 2/2 | 1/3 | 12/27 |
|  | Deletion not involving *CACNA1A* | |  |  |  |  | 0/2 | 2/3 |  | - |  |  | 1/1 |  |  |  |  | + |  |  |  | 4/8 |

**Legend:** F: female; M: male; +: present; -: absent; NA: not available; OFC: occipital frontal circumference; GDD: global developmental delay
